# Interactions between Neutrophils and *Pseudomonas aeruginosa* in Cystic Fibrosis

**DOI:** 10.3390/pathogens6010010

**Published:** 2017-03-09

**Authors:** Balázs Rada

**Affiliations:** College of Veterinary Medicine, Department of Infectious Diseases, The University of Georgia, 501 D.W. Brooks Drive, Athens, GA 30602, USA; radab@uga.edu; Tel.: +1-706-5423695; Fax: +1-706-5425771

**Keywords:** *Pseudomonas aeruginosa*, cystic fibrosis, neutrophil, neutrophil extracellular traps, biofilm, flagellum, phagocytosis, oxidative, killing

## Abstract

Cystic fibrosis (CF) affects 70,000 patients worldwide. Morbidity and mortality in CF is largely caused by lung complications due to the triad of impaired mucociliary clearance, microbial infections and chronic inflammation. Cystic fibrosis airway inflammation is mediated by robust infiltration of polymorphonuclear neutrophil granulocytes (PMNs, neutrophils). Neutrophils are not capable of clearing lung infections and contribute to tissue damage by releasing their dangerous cargo. *Pseudomonas aeruginosa* is an opportunistic pathogen causing infections in immunocompromised individuals. *P. aeruginosa* is a main respiratory pathogen in CF infecting most patients. Although PMNs are key to attack and clear *P. aeruginosa* in immunocompetent individuals, PMNs fail to do so in CF. Understanding why neutrophils cannot clear *P. aeruginosa* in CF is essential to design novel therapies. This review provides an overview of the antimicrobial mechanisms by which PMNs attack and eliminate *P. aeruginosa*. It also summarizes current advances in our understanding of why PMNs are incapable of clearing *P. aeruginosa* and how this bacterium adapts to and resists PMN-mediated killing in the airways of CF patients chronically infected with *P. aeruginosa*.

## 1. Antimicrobial Mechanisms of Neutrophils against *Pseudomonas aeruginosa*

### 1.1. *P. aeruginosa*, an Opportunistic Pathogen

*Pseudomonas aeruginosa* has a widespread occurrence in aqueous environments in nature [1,2]. This ubiquitous, Gram-negative pathogen has a large genome that enables its adaptation to diverse growth conditions and infections in many species, ranging from nematodes to vertebrates, including humans [3,4]. *P. aeruginosa* uses several virulence factors to establish infection [3]. *P. aeruginosa* can be found in the environment in two main, different growth forms, as planktonic bacteria and biofilms [5]. Planktonic bacteria harbor several surface appendages that mediate motility (flagellum, pilus) and virulence (secretion systems) [6,7]. *P. aeruginosa* residing in biofilms becomes metabolically less active and more resistant to attacks of the immune system or any medical treatment [8,9,10]. The medical relevance of *P. aeruginosa* biofilms is so high that this bacterium became the model organism for microbial biofilm research. While this review focuses on interactions between *P. aeruginosa* and neutrophils, several excellent reviews provide deeper insights into different aspects of *P. aeruginosa* biology.

Healthy humans are protected against *P. aeruginosa* and do not typically suffer from infections caused by this bacterium. Immunocompromised individuals or patients with deficient clearance mechanisms are the ones mainly infected by *P. aeruginosa*. *P. aeruginosa* is an opportunistic pathogen posing a significant medical problem to society. *P. aeruginosa* is one of the main leading causes (18%–20%) of nosocomial lung infections (also called hospital-acquired or health care-associated pneumonia) [11,12,13,14]. This is due to the high prevalence of immunocompromised patients, biofilm growth of *P. aeruginosa* on plastic surfaces of medical devices, increased occurrence of multidrug-resistant strains in hospital wards and healthcare personnel carrying this bacterium [15,16,17,18]. Neutropenic patients are immunocompromised and also frequently infected with *P. aeruginosa* [19]. Chemotherapy of cancer patients often results in neutropenia and *P. aeruginosa* lung infections [20,21]. Susceptibility of human immunodeficiency virus (HIV) patients to *P. aeruginosa* is also high; 8%–25% of HIV patients with pneumonia are infected with this bacterium [14]. Chronic obstructive pulmonary disease (COPD) patients are also frequently colonized in their lungs with *P. aeruginosa* (4%–15% of adult COPD patients) and present diverse symptoms ranging from mild bronchitis to pneumonia with sepsis [22,23,24].

In addition to the transient infections mentioned above, *P. aeruginosa* can also establish persistent lung infections, such as in patients with cystic fibrosis (CF), non-CF bronchiectasis and primary ciliary dyskinesia (PCD). Although more prevalent than CF, less is known about the diverse etiologies of non-CF bronchiectasis. Non-CF bronchiectasis patients typically develop permanent damage and dilation of the lower airways due to prior pneumonia and become infected with organisms, like *P. aeruginosa*, despite any obvious abnormalities in their immune system [24]. PCD is characterized by impaired mucociliary clearance of the airways that prevents mucus transport and enables persistent infections with bacteria, including *P. aeruginosa* [25]. Although *P. aeruginosa* infects the lungs of human patients with a variety of conditions, the disease most tightly associated with this bacterium is CF.

### 1.2. Cystic Fibrosis

Cystic fibrosis is a common genetic disease affecting 70,000 people worldwide [26]. Mutations in the cystic fibrosis transmembrane regulator (CFTR) anion channel alter normal ion and fluid transport across the airway epithelium, lead to thickened mucus formation, impaired mucociliary clearance, bacterial adherence and inflammation [26,27]. The vicious cycle of impaired clearance-infection-inflammation drives long-term lung damage, bronchiectasis, airflow obstruction and death in CF [27]. In the lungs, CFTR is primarily expressed in epithelial cells, and this deficiency is the primary cause of lung disease. It is still debated whether mucociliary clearance enables microbial colonization first that drives subsequent inflammation or the epithelial CFTR defect triggers a hyperinflammatory phenotype prior the appearance of any microbes. The recently-developed pig animal model of CF lung disease started to clarify some of these unanswered questions [28]. Although CF lungs host polymicrobial infections, *P. aeruginosa* is one of the major pathogens infecting a large part of CF patients [26,27,29]. According to the 2015 Cystic Fibrosis Foundation Patient Registry Annual Data Report, while *P. aeruginosa* used to be the most prevalent microorganism infecting CF patients, its prevalence continues to decrease. On the other hand, *Staphylococcus aureus* prevalence is increasing. These trends could be partially due to aggressive, early strategies to eradicate *P. aeruginosa* and more sensitive methods to detect Gram-positive microorganisms. Chronic *P. aeruginosa* infection has been linked to more rapid progression of lung disease and mortality in CF [30,31,32]. *P. aeruginosa* infects CF patients early in life and becomes a persistent pathogen in subsequent years [33]. Its persistence in the CF lung is thought to be due largely to its ability to form biofilms [34,35,36]. In biofilms, *P. aeruginosa* bacteria are embedded in a self-produced polymer matrix mainly consisting of the polysaccharide alginate [36]. *P. aeruginosa* biofilms also contain self-produced or foreign DNA in CF airways [36]. Biofilms protect bacteria against attacks of the immune system or antibiotic treatments and provide the advantage to survive in the environment of the CF lung.

**Figure 1:**
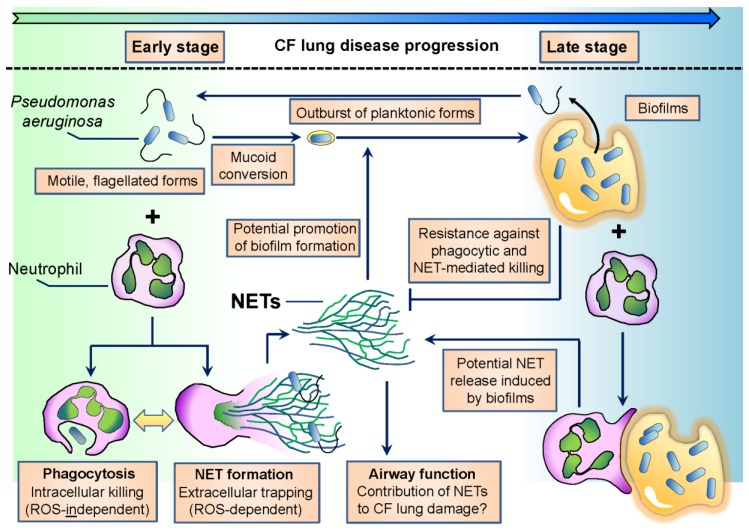
Scheme of complex interactions between polymorphonuclear neutrophil granulocytes (PMNs) and *Pseudomonas aeruginosa* in cystic fibrosis (CF) airways with a special emphasis on neutrophil extracellular traps (NETs). ROS: reactive oxygen species.

Early isolates of *P. aeruginosa* in CF are planktonic, characterized by high motility and flagellum expression [37] (Figure 1). The flagellum is crucial for *P. aeruginosa* to establish infection in several hosts since flagellum-deficient strains are severely reduced in virulence [38,39]. On the other hand, over the course of CF infection, one of the most characteristic changes in *P. aeruginosa* phenotype is the loss of flagellar motility [33,37] (Figure 1). Adaptation of *P. aeruginosa* to airways in chronic CF also involves mucoid conversion, alginate production, a decrease in virulence factor expression and biofilm formation [37]. Culturing *P. aeruginosa* in CF sputum results in similar changes [40]. The flagellum is also needed to initiate biofilm formation by *P. aeruginosa* [41,42]. In chronic stages of CF airway disease, *P. aeruginosa* is dominantly found in the form of biofilms. Biofilm cultures are resistant against the attacks of the immune system and medical treatments, including antibiotics, and ensure long-term survival of the bacterium in the host. Several excellent reviews deal with the details of the formation, regulation and structure of *P. aeruginosa* biofilms [8,43,44,45,46].

It is of high clinical relevance to understand how *P. aeruginosa* can persist in CF lungs for years and why it is not efficiently eliminated by the immune system, particularly neutrophils that are present in large numbers and drive inflammation [31].

### 1.3. Neutrophils Are Key to Eliminate *P. aeruginosa*

*P. aeruginosa* is a frequent pathogen in neutropenic patients irrespective of the cause of low PMN numbers (chemotherapy, HIV) [14,20,21]. Humans deficient in key neutrophil-mediated antimicrobial mechanisms, such as specific granule deficiency or leukocyte adhesion deficiency, are prone to *P. aeruginosa* infection [47]. PMN depletion in mice leads to enhanced mortality in acute *P. aeruginosa* lung infection models [48,49]. In neutropenic mice, a 10^5^-times smaller dose of *P. aeruginosa* is enough to lead to the same lethal effect in a lung infection model than in mice with normal PMN numbers [48]. Similar effects were shown in rabbits, as well [50]. Airway epithelial control of PMN infiltration has been shown to be crucial to fight *P. aeruginosa* in murine airways [51,52]. The antimicrobial peptide LL-37, highly expressed in PMNs, also augments *P. aeruginosa* clearance [53,54]. Overall, PMNs play a critical role in fighting *P. aeruginosa*.

### 1.4. PMN Recruitment to the Airways

The appearance of *P. aeruginosa* in mammalian airways is always accompanied by robust PMN infiltration that is driven by chemoattractant molecules primarily released by airway epithelial cells or already recruited leukocytes, including PMNs. Interleukin 8 (IL-8) is one of the most potent PMN-recruiting chemokines that can be released by epithelial cells, PMNs and macrophages and is recognized by two chemokine receptors, CXCR1 and CXCR2 (C-X-C motif chemokine receptor 1 and 2) [55]. Concentrations of IL-8 in CF airways is elevated and correlated with lung damage [56,57,58,59]. Elevated sputum IL-8 levels also correlated with *P. aeruginosa* infection in a cohort of CF patients [60]. IL-8 is also detected in the exhaled breath condensate of CF patients, and it is elevated in patients infected with *P. aeruginosa* [61]. PMNs and macrophages can be major sources of IL-8 in CF since both phagocytes release IL-8 in response to both microbial lipopolysaccharide (LPS) and host stimuli (IL-1β, TNF-α) [62]. Airway epithelial cells release IL-8 in response to *P. aeruginosa*, LPS or its extracellular pigment pyocyanin [63,64,65,66]. PMN elastase present in CF airway fluids has also been shown to induce IL-8 release in airway epithelial cells [67]. In addition to IL-8 release stimulated by exogenous microbial or host stimuli, enhanced endogenous IL-8 production has also been proposed to drive early inflammation in CF. CFTR-deficient airway epithelial cells secrete larger amounts of IL-8 than their wild-type counterparts, indicating an epithelium-derived proinflammatory effect of IL-8 in CF [68,69,70]. Endoplasmic reticulum stress and exaggerated NF-κB activation induced by misfolded CFTR is the likely reason for enhanced chemokine production in CF epithelial cells [71,72,73]. Among the many potential PMN-recruiting molecules present in the CF sputum [74], the complement activation product C5a has been shown to be important to optimal mucosal defense against *P. aeruginosa* in the murine lung [75]. Formation of C5a has been demonstrated in CF airway fluids [76]. Whether C5a is a main PMN recruiter in CF airways remains to be elucidated as PMN serine proteases were suggested to degrade and inactivate the C5a receptor on PMNs [77]. Although not as potent as the previous molecules, the proinflammatory cytokine IL-1β can also direct PMN migration. Elevated levels of IL-1β, a proinflammatory cytokine produced by inflammasomes, have been detected in CF airways and correlated with lung damage [59,78]. Although CF macrophages harbor an intact inflammasome capable of releasing IL-1β in response to *P. aeruginosa*, it remains to be better understood whether enhanced IL-1β levels seen in CF are due to intrinsic increase in NF-kB activation [79]. Although on a per cell basis, PMNs release far less IL-1β than macrophages, considering the total amount of PMNs in CF airways, PMNs could be a pathologically relevant source of IL-1β in CF [80]. The lipid leukotriene B4 is a product of the arachidonic acid metabolic pathway, released by leukocytes, including PMNs, and one of the most potent molecules driving PMN chemotaxis. Leukotrienes are present in CF sputum [81,82,83,84]. CF PMNs were shown to have decreased chemotaxis in response to LTB4 compared to non-CF PMNs, while their chemotactic response to formyl peptides was the same [85]. Leukotriene B4 is also detectable in exhaled breath condensates of CF patients and is elevated upon *P. aeruginosa* infection [61]. IL-17, a potent PMN-recruiting cytokine produced by Th_17_ cells, is essential to prevent chronic *P. aeruginosa* infection in mice [86]. CF airways have elevated levels of IL-17 [61,87]. IL-23, a cytokine closely related to IL-17, is essential to protect mice from *P. aeruginosa* lung infection [88]. IL-17 immunolocalizes to PMNs and monocytes in CF airways [89,90]. Based on elevated levels of IL-17 in CF and the essential role of the IL-17/IL-23 axis in regulating PMN recruitment, it has also been proposed that CF is a Th_17_ disease [91,92].

In addition to host molecules, pathogen-associated molecular patterns (PAMPs) can also be strongly chemotactic for PMNs, but their presence in CF airway fluids and contribution to PMN recruitment are less clear. Formyl peptides shedding from bacteria are sensed by PMNs in lower nanomolar concentrations by two different receptors, FPR1 and FPR2 [93]. Formyl peptides have been found in CF sputum [74]. *P. aeruginosa* flagellin is detectable in the airways of chronic CF patients [94] that is known to stimulate IL-1β release in epithelial cells and macrophages [95]. The *Pseudomonas* quinolone signal (PQS) that is a quorum sensing molecule of *P. aeruginosa* has been shown to recruit PMNs via the MAPkinase p38, while it did not affect bactericidal function or induce apoptosis [96].

Thus, PMNs are recruited to CF airways by several mechanisms [74]. It remains to be elucidated whether any one of the several PMN chemoattractants plays a major role and could be targeted therapeutically. 

Overall, PMNs provide the most efficient branch of the immune system to fight *P. aeruginosa* lung infections.

### 1.5. Intracellular Killing Following Phagocytosis

What mechanisms are used by PMNs to eliminate *P. aeruginosa* in healthy hosts? The most efficient way of eliminating *P. aeruginosa* by PMNs is classical phagocytosis and subsequent intracellular killing. PMNs are highly phagocytic leukocytes capable of rapidly engulfing up to 10–20 bacteria per cell in a short period of time [97]. Bacteria first have to be recognized by PMNs. PMNs are equipped to bind to several PAMPs expressed on the surface of bacteria via their pattern-recognition receptors (PRRs). C-type lectins, among them Dectin-1 especially, are important phagocytic PRRs expressed on PMNs essential for uptake and elimination of fungal pathogens [98]. Another PRR, TREM-1, recognizes several microbes and mediates their phagocytosis in PMNs [99]. NOD1 and NOD2 are nonphagocytic, intracellular PRRs binding cytosolic microbial ligands [97,100]. NOD1 participates in the immune response against *P. aeruginosa* [101]. The nonphagocytic Toll-like receptors (TLRs) recognize a wide range of microbial PAMPs (DNA, RNA, peptides, lipids and carbohydrates) and prime PMNs for enhanced effector responses, including phagocytosis [100]. Microbial uptake of PMNs is several times accelerated when bacteria have been labelled by the immune system. This process called opsonization ensures faster recognition and killing of microbes and is especially important for fighting pathogens that are not recognized directly by PMNs. Classical opsonins include immunoglobulin G (IgG) and the complement activation product C3b. PMNs express two complement receptors prior to activation, CR3 and CR4, both binding to C3bi [100]. Upon stimulation, PMNs mobilize another opsonin receptor, CR1 to their surface recognizing C3b/C4b complexes further enhancing their capacity to engulf microbes [100]. Mannan-binding lectin is a soluble PRR that binds C1q on the microbial surface and promotes phagocytosis in PMNs by binding to the C1q receptor [100]. IgGs are recognized by PMNs via low and high affinity Fc receptors, FcRs [102]. The low affinity FcRs, FcγRIIA and FcγRIIIB, are expressed in PMNs prior to activation, while the surface expression of the high affinity FcγRI on PMNs is increased upon microbial stimulation [100]. Pentraxins form a phylogenetically old group of pentameric plasma proteins that recognize bacterial or fungal PAMPs and serve as the third major type of opsonin receptors [102]. *P. aeruginosa* is sensitive against the action of pentraxin PTX3 [103], and the risk of *P. aeruginosa* airway colonization in CF patients is affected by PTX3 genetic variations [104].

*P. aeruginosa* can be phagocytosed by PMNs similarly to several other microbes [102]. Both FcRs and CR3 have been implicated to participate in opsonic phagocytosis of *P. aeruginosa* (for details, see [103]). An early report indicated the involvement of CD14 in *P. aeruginosa* nonopsonic phagocytosis [104]. TLRs likely do not directly mediate nonopsonic uptake since deficiency in MyD88 does not impair mouse macrophage phagocytosis of *P. aeruginosa* [105]. Very likely, several receptors and their activation pathways are responsible for optimal phagocytosis of *P. aeruginosa* [103].

Phagosomes containing *P. aeruginosa* fuse intracellularly with PMN granules storing an arsenal of antimicrobial molecules (detailed in [106]). The phagolysosome fusion creates a special, confined niche in which bacteria can be eliminated efficiently in a precise surgical way without significant leaking of the dangerous PMN cargo into the environment [100]. Four types of PMN granules have been categorized and referred to as primary (or azurophil), secondary (or specific), tertiary (or gelatinase) granules and secretory vesicles [106]. Secretory vesicles are membrane vesicles derived from the plasma membrane primarily containing plasma membrane proteins and extracellular milieu [106]. Primary, secondary and tertiary granules have specific contents and fuse with the phagosome in a reverse order, tertiary granules first and primary granules last [106]. Granule content can also be released into the extracellular milieu when the granule membrane fuses with the plasma membrane via a process called degranulation [106]. Extracellular mobilization of secretory vesicles and gelatinase granules can be achieved relatively easily in PMNs upon weak stimulation, while primary and secondary granules are only released in the extracellular medium when they leak during phagocytosis [106].

PMNs possess several antimicrobial mechanisms that contribute to microbial killing in the phagolysosome. The production of reactive oxygen species (ROS) is a hallmark of PMN activation. ROS comprise short-lived, reactive molecules, ions and radicals that are all derivatives of molecular oxygen [107]. The phagocytic NADPH oxidase expressed in PMNs produces superoxide anions from molecular oxygen as the primary type of ROS [108]. Superoxide can dismutate to hydrogen peroxide (H2O2) spontaneously or with the help of the superoxide dismutase enzyme [108]. Formed H2O2 will be used by myeloperoxidase (MPO), a peroxidase present in large quantities in primary granules of PMNs, to produce highly reactive hypochloric acid (HOCl) [108]. Further, ROS that can be produced by PMNs involve singlet oxygen, hydroxyl radical, chloramines and peroxynitrites [107,108]. ROS are thought to damage microbes primarily by direct chemical attack, but an alternative hypothesis has also been proposed that ROS-driven ion fluxes are actually necessary for optimal phagosomal digestion [97,109,110]. PMNs harbor several antimicrobial products that attack microbes in an ROS-independent manner. Primary granules contain bacterial permeability increasing protein (BPI), neutrophil elastase (NE), proteinase 3, cathepsins, defensins and lysozyme, just to name a few [106]. BPI is a pluripotent protein that neutralizes bacterial LPS, promotes the delivery of bacterial outer membrane antigens to dendritic cells and has antibacterial activity against *P. aeruginosa* [111,112]. Neutrophil elastase, cathepsins and proteinase 3 are serine proteinases whose functions are to degrade the extracellular matrix and to attack microbial proteins [113]. NE-deficient mice are more susceptible to *P. aeruginosa* lung infection than their wild-type counterparts [114], while cathepsin G-deficient animals have lower bacterial lung burden [115]. Defensins are antimicrobial peptides released from PMNs in large amounts that kill *P. aeruginosa* efficiently [116]. Lysozyme treatment ameliorates *P. aeruginosa* pneumonia [117,118]. LL-37, an antimicrobial peptide belonging to the cathelicidin family, is highly expressed in PMNs, stored in secondary granules and efficient in attacking *P. aeruginosa* [53]. Thus, PMNs are well-equipped with redundant antimicrobial mechanisms to fight diverse pathogens.

### 1.6. Neutrophil Extracellular Traps

In addition to intracellular killing [119], PMNs also trap and kill extracellular microbes via another mechanism referred to as neutrophil extracellular trap (NET) formation [120]. NETs are composed of a DNA scaffold associated with histones and neutrophil granule components, such as MPO and NE [120,121,122]. NET formation has been originally proposed to be an active way of cell death involving cytoskeletal changes, plasma and nuclear membrane disintegration and DNA extrusion [120,123]. Later on, alternative mechanisms of NET release were discovered that do not require cell death, occur much faster and include mitochondrial DNA [124]. Several microorganisms including *P. aeruginosa* were shown to trigger NET release in PMNs. Only NET-forming PMNs release protein-DNA complexes (myeloperoxidase-DNA or neutrophil elastase-DNA); not apoptotic or necrotic PMNs [120,125,126,127,128]. Despite the vast amount of literature published in the last 12 years on NETs, signaling pathways leading to NET formation are still largely unknown, and only the involvement of a few molecules is known. The NADPH oxidase has been shown to be required for NET release early on since PMNs isolated from chronic granulomatous disease (CGD) patients with nonfunctional NADPH oxidase did not form NETs in response to different microbes [123,129]. Residual NADPH oxidase activity in CGD has been indicated to determine PMNs’ ability to release NETs [130]. Similarly, MPO, NE and the citrullination of histones mediated by peptidylarginine deiminase 4 (PAD4) were also shown to be required for NET formation [131]. Citrullinated histones are only present in NETs, not in resting PMNs [132]. PAD4-deficient murine PMNs do not form NETs in vitro [133,134], and PAD4-deficient mice have impaired NET-mediated antibacterial defenses [135]. The fact that the purpose of NET formation is extracellular trapping of microbes by PMNs to help localize the infection is well accepted. It remains though controversial whether NETs are also efficient in killing trapped microorganisms [136]. Extracellular DNA (ecDNA) has been shown to be antimicrobial against *P. aeruginosa* in in vitro studies [137,138]. EcDNA itself is thought to scavenge large amounts of cations and thereby limit microbial growth [137,138]. NET-associated antimicrobial proteins and peptides were suggested to harm trapped microorganisms [120]. This has been challenged by questioning whether the extracellular concentration of NET-related enzymes and proteins is sufficient to support such a killing effect [139]. NET-linked myeloperoxidase was shown to kill microorganisms in vitro in the presence of hydrogen peroxide [140]. The exact microbicidal potential of NETs, especially in vivo, remains to be elucidated.

In addition to the beneficial antimicrobial role of NETs, several studies suggested their contribution to the pathology of numerous conditions, including CF lung disease. NETs were shown to drive or contribute to the pathology of numerous autoimmune diseases, such as systemic lupus erythematosus, rheumatoid arthritis and small-vessel vasculitis [141]. In gout, a dual pro- and anti-inflammatory role has been proposed for NETs in disease pathogenesis [142]. NETs have also been linked to cancer, diabetes and viral infections [143,144,145]. Although NETs are currently the most studied field of PMN biology, several questions need to be answered with regard to their roles in antimicrobial defenses and clinical manifestation of several diseases.

### 1.7. PMN Microvesicles

The most recently-described mechanism by which PMNs attack microbes is the release of microvesicles [146]. Vesicle (diameter 100–1000 nm) shedding from live cells obtained significant attention is the past few years as a novel tool of cell-to-cell communication [147]. They are involved in antigen presentation, the transfer of receptor proteins or RNA and host defense. PMNs also release microvesicles that have in vitro antimicrobial activity against *S. aureus* [146]. The amount of PMN-derived microvesicles increases robustly during inflammatory conditions [148]. A diverse set of host and microbial stimuli was shown to induce microvesicle release from PMNs [148]. In addition to direct microbial killing, PMN-derived microvesicles were also shown to modulate the function of other immune cells [148]. Although a new area of research, PMN-derived microvesicles represent an exciting field with potential future implication to host defense, autoimmunity and immunomodulation.

### 1.8. Oxidative and Non-Oxidative PMN Mechanisms against *P. aeruginosa*

The PMN antimicrobial mechanisms highlighted above can also traditionally be classified as oxidative and non-oxidative, based on their dependence on NADPH oxidase-produced ROS. *P. aeruginosa* is fairy resistant against the oxidative mechanisms and is mainly susceptible to non-oxidative actions of PMNs. This is well-supported by human clinical data showing that patients with impaired or completely absent PMN respiratory burst (CGD, myeloperoxidase-deficiency) do not suffer from *P. aeruginosa* infections [149,150,151]. This indicates that the NADPH oxidase or myeloperoxidase are not required to eliminate *P. aeruginosa* from healthy hosts, no matter what their contribution to bacterial killing is. CGD PMNs kill *P. aeruginosa* efficiently in vitro, further supporting the previous conclusion [152]. *P. aeruginosa* catalase has been proposed to be responsible for its resistance against oxidative PMN killing mechanisms [152]. *P. aeruginosa*-induced ROS production in PMNs could in fact support long-term survival of the bacterium since hydrogen peroxide was shown to promote mucoid conversion of *P. aeruginosa* [153]. On the other hand, several molecules of the non-oxidative PMN antimicrobial repertoire are efficient tools to kill *P. aeruginosa*: NE, lysozyme, defensins and cathelicidins [53,114,116,117,118]. Thus, reducing oxidative and promoting non-oxidative PMN antimicrobial mechanisms holds the best promise to achieve significant improvements in *P. aeruginosa*-infected patients by targeting PMNs. Whether this could work in CF remains to be determined since CF patients are infected with other microbes. *S. aureus*, another main pathogen in CF airways [154], is mainly killed by oxidative mechanisms of PMNs [97] and is also a main pathogen in CGD [155].

In summary, PMNs possess several antimicrobial tools whose optimal coordination in time and space is required for efficient elimination of microbes involving *P. aeruginosa*. If mistakes occur, PMN activation causes host damage with potential, serious, clinical consequences. Therefore, it is essential to understand which mechanisms of PMNs are the most efficient to attack pathogens and how to avoid PMN-mediated pathology occurring in several diseases, including CF.

## 2. PMN Dysfunction in CF

Airway inflammation in CF is the product of a complex set of innate immune interactions. The PMN is a pivotal cellular player influencing the outcome of these interactions. CF airways contain large numbers of PMNs that fail to successfully eliminate bacteria and cause lung damage. The main points of PMN dysfunction in CF are listed in this section with cited literature that provides more detailed insights into each direction.

### 2.1. PMN Components Correlate with CF Lung Disease Severity

PMN density in CF sputum correlates with CF lung disease severity (measured as forced expiratory volume in one second, FEV_1_) [57,78]. Sputum and blood concentrations of NE and MPO in CF patients are associated with declines in lung function [57,58,78,156,157]. NE is a major risk factor for bronchiectasis in CF children [158,159]. Sputum NE levels have been shown to be the best predictors of CF lung function decline so far [158,160]. Improper diminishment of inflammation during an exacerbation is linked to failure to recover respiratory function and increased risk of subsequent re-exacerbation in patients with CF [161]. IL-8, a major PMN chemoattractant that both airway epithelial cells and PMN themselves produce, has also been associated with CF lung function decline [57,58]. Interlekuin-1β (IL-1β), a proinflammatory cytokine recruiting PMNs mainly produced by macrophages, but also secreted by activated PMNs, has also been linked to CF lung damage [78]. These data obtained from clinical samples of CF patients clearly show that PMNs are the clinically most important leukocyte in CF airways, and PMN-mediated inflammation contributes to lung disease.

### 2.2. CFTR Deficiency in PMNs

CF is a genetic disorder potentially affecting all cells expressing CFTR. PMN functions could be also primarily affected by CFTR deficiency. Since the first detection of CFTR in PMNs [162], reports described impaired intracellular killing of *P. aeruginosa*, diminished chloride transport, reduced cytosolic calcium changes, altered response to *N*-formyl-methionyl-leucyl-phenylalanine and diminished degranulation in human or murine CFTR-deficient PMNs [163,164,165,166,167,168,169]. On the other hand, NET formation of CF PMNs and their intrinsic superoxide production was not different from that of non-CF PMNs [170,171]. Concerns about the in vivo relevance of primary CFTR deficiency in PMNs in CF are raised by indirect clinical observations. In CF, PMN dysfunction has been reported and widely accepted only in one organ, the lung. If CFTR were to affect essential antimicrobial functions of PMNs in vivo, the involvement of several other organs would be expected, as is seen in PMN-specific genetic disorders, such as CGD [150,172]. Another possibility is that CFTR deficiency in PMNs can be overcome by parallel mechanisms in each organ, except for the lung. An important argument for PMN-mediated lung damage in CF is the fact that CFTR-deficient airway epithelial cells are major drivers of PMN recruitment. Although exciting results are emerging with regard to the direct or indirect mechanisms by which CFTR affects PMN functions in CF, future research using novel tools like a PMN-specific conditional CFTR knockout mouse strain could help better answer this question. Several excellent reviews provide a deeper insight into this field [173,174,175].

### 2.3. CF Airway PMNs

A neglected, but clinically very important area of research is the investigation of PMNs found in the airways of CF patients. Studies by a few groups described that CF airway PMNs are different from those found in the blood of the same CF patient [176,177,178,179,180,181,182]. PMNs in CF airways have been shown to have enhanced expression of certain metabolic and stress pathways including CD39, CXCR4, CD114 and RAGE [177]. It has been suggested that a shift in the expression of their nutrient transporters affecting glucose and inorganic phosphate transports is part of their adaptation to the CF airway environment [176]. CF airway PMNs have a reduced respiratory burst and altered expressions of TLRs [179,182,183]. Although these cells are present at the site of infection and drive CF airway inflammation, our knowledge about them is limited at this point. Future studies will have to determine whether their potentially altered metabolic and antimicrobial phenotypes are due to CFTR deficiency, factors found in CF airways, in the tissues surrounding them during migration, or a combination of all of them.

### 2.4. NETs in CF

The presence of large amounts of PMNs in CF airways has been detected early on, but the mechanism by which PMNs discharge their DNA and granule content still remains uncertain even today. PMNs were thought to die in CF by apoptosis followed by necrosis due to neglect (lack of removal of apoptotic cells, secondary necrosis). PMNs did not form the focus of CF research for a long time partially because successful pharmaceutical targeting of a necrotic PMN death pathway seemed very unlikely. In addition, PMN-driven inflammation might have had dual roles in CF airways: PMNs likely fulfil antimicrobial functions while they also contribute to lung damage. Before clearly understanding the exact role of PMNs in CF airway disease, targeting them in CF is problematic.

With the discovery of NETs, a new, alternative explanation for PMN dysfunction in CF emerged [120]. By releasing NETs, CF airway PMNs would respond to the presence of bacteria, but at the same time would also release their DNA and granule content. EcDNA is present in large amounts in CF airways, and DNAse therapy has been used since the 1980s. Although DNAse therapy improves lung function of CF patients [184,185,186,187] and has a remarkable safety profile over several decades, its effect is not universal [188], does not completely degrade ecDNA [189] and does not improve lung inflammation [186]. ecDNA in CF airways is derived from the host [190], mainly PMNs [78,156,190]. NETs are abundantly present in CF airways [191,192]. Negative correlations were found between CF sputum ecDNA concentrations and lung function measures [58,193]. Neutrophils undergoing NET formation were detected in CF sputum samples [191,192,194]. Histone citrullination, a histone modification characteristic for NET formation, but not for neutrophil apoptosis or necrosis, was also detected in CF sputum [191]. Despite the abundance of NETs in CF airways, NETs have not yet been quantitatively correlated with CF airway disease, it remains unknown what are the main stimuli of NET formation in CF, and what is the molecular-cellular mechanism of NET release by CF airway PMNs.

### 2.5. Anti-Inflammatory Strategies in CF

Although we do not completely understand the details of PMN-mediated inflammation in CF, anti-inflammatory strategies have been tested and are being used to improve CF lung disease. While several excellent reviews provide deeper insight into this field, here, only the main approaches will be mentioned [195,196,197,198]. Inhibition of NE represents a rather classical approach to diminish PMN-mediated lung damage in CF [199,200,201]. Phosphodiesterase (PDE4) inhibitors reduce cAMP synthesis and thereby have direct inhibitory action on inflammatory cell signaling and PMN recruitment [195]. Attractive lipid mediator targets are lipoxins, mainly LXA4, that are generated from arachidonic acid and have a general suppressive role on inflammation, including PMN effector mechanisms [195]. Resolvins are other lipid mediators of interest because they also have general anti-inflammatory, pro-resolution effects, including inhibition of PMN respiratory burst, chemotaxis and attachment [195]. The cannabinoid receptor CB2 is highly expressed on immune cells, and its activation promotes anti-inflammatory effects, including reduced proinflammatory cytokine production and leukocyte migration [195]. Leukotriene modulators target two enzymes, 5-lipoxygenase and leukotriene A4 hydrolase, in the hope of significantly reducing LTB4 levels and preventing PMN recruitment [195]. As mentioned above, Th17 cells play an important role in orchestrating PMN recruitment to the airways. Targeting IL-17 with therapeutic monoclonal antibodies works in asthma and autoimmune diseases and represents a novel, exciting approach to inhibit PMN recruitment and inflammation in CF [202,203].

In summary, PMNs in CF fail to eliminate respiratory pathogens, including *P. aeruginosa*. PMNs could provide a promising and most likely powerful target for anti-inflammatory pharmaceutical intervention. PMN targeting in CF remains infeasible until we clearly understand their contribution to antimicrobial defense and lung damage.

## 3. Adaptation of *P. aeruginosa* to Neutrophil-Mediated Attacks in CF

*P. aeruginosa* is a major pathogen infecting the airways of CF patients [37]. *P. aeruginosa* is not cleared from CF airways in spite of being surrounded by several PMNs. This is likely due to its successful adaptation to the environment found in the CF lung including PMNs. In this section, the mechanisms are discussed that are potential contributors to *P. aeruginosa* adaptation against PMN-mediated effector mechanisms in CF.

### 3.1. Loss of Flagellar Motility

As mentioned previously, early isolates of *P. aeruginosa* are motile. Motile bacteria are easy to recognize by the immune system, and retaining a motile flagellum would lead to rapid microbial clearance. Phagocytes (macrophages) much more likely recognize and phagocytose flagellated *P. aeruginosa* than its aflagellated counterpart [103,204] (Figure 1). PMNs also phagocytose *P. aeruginosa* in a flagellum-dependent manner [205]. Impaired phagocytosis of flagellum-deficient *P. aeruginosa* is due to the loss of its motility, not flagellum expression [105]. Chronic CF isolates of *P. aeruginosa* are typically nonmotile and resist phagocytic clearance of macrophages [206]. It has been presumed that host-selective pressure inspires *P. aeruginosa* to lose its motility in chronic CF, especially in patients with poor clinical conditions [207,208]. The flagellum is also required for *P. aeruginosa*-induced superoxide formation and NET release in human PMNs [205]. Lack of flagellar motility, not flagellum expression per se, is responsible for impaired NET release induced by flagellated *P. aeruginosa* [205]. This is consistent with previous data showing that early CF isolates of *P. aeruginosa* induce larger amounts of NETs and are more resistant against NET-mediated attacks than late isolates obtained from the same CF patients [128,170] (Figure 1). Whether loss of swimming motility alone is responsible for reduction in *P. aeruginosa*-initiated NET release or loss of other NET-inducing or appearance of NET-inhibitory factors also contribute, remains to be studied. PMN products have been shown to attack *P. aeruginosa* flagellin. NE degrades bacterial flagellin and represses its transcription [209,210]. Lactoferrin, a PMN antibacterial product, attacks flagellin-dependent biofilm formation of *P. aeruginosa* [211]. Extrusion of NETs induced by motile *P. aeruginosa* must primarily occur in early disease stages characterized by flagellated bacterial forms (Figure 1). Its occurrence in chronic CF is, however, also likely since *P. aeruginosa* flagellin has been detected in sputa of CF patients chronically infected with *P. aeruginosa*, independently of the presence of mucoid bacterial strains [94]. Planktonic, flagellated forms of *P. aeruginosa* break out from biofilms in the environment, and this could also happen in chronic CF airways (Figure 1).

Overall, flagellum-provided motility is essential to establish infection in CF airways, but its downregulation is required to avoid immune recognition and to enable long-term bacterial adaptation to the host in chronic infection. PMNs likely represent one of the main factors of the CF airway environment responsible for the described changes in *P. aeruginosa* motility (Figure 1).

### 3.2. Characteristic Image of Chronic CF Airways: Suspension Biofilms of *P. aeruginosa* Surrounded by PMNs

Laboratory biofilms of *P. aeruginosa* have been studied for a long time. In chronic CF airways, *P. aeruginosa* does not form; however, these flat, thick biofilms covering the airway surface but reside instead in three-dimensional biofilms also called suspension biofilms or non-attached aggregates [212,213,214,215,216]. These bacterial aggregates accompanied by large amounts of PMNs represent the typical clinical image found in the airways of CF patients chronically infected with *P. aeruginosa* (Figure 1) [212,213,214,215,216].

This mode of biofilm growth is likely not unique to CF airways as *P. aeruginosa* has been reported to form similar aggregates under different stress conditions, in the absence of any mammalian cells. The *P. aeruginosa* PAO1 strain was grown in the presence of the detergent sodium dodecyl sulfate, and macroscopic aggregates consisting of respiring bacterial cells embedded in an extracellular matrix composed of DNA and acidic polysaccharides formed [217]. Aggregated bacteria showed a significantly increased rate of survival over planktonic cells in the presence of the detergent [217]. Starvation of *P. aeruginosa* results in similar aggregate cultures in liquid medium that also contains extracellular DNA originating from bacteria [218,219,220]. Phenazines induce DNA release from *P. aeruginosa* via H2O2 generation [221,222]. The structure of the suspension biofilms is similar to those of the laboratory biofilms [220]. Important roles of calcium and c-di-GMP signaling in supporting suspension biofilm growth of *P. aeruginosa* have just been revealed [223,224]. Bacterial growth rates inside of *P. aeruginosa* suspension aggregates negatively correlated with the size of these aggregates in the CF lung [213]. Quorum sensing molecules, PQS and AHL-12, produced at early stages of biofilm formation of *P. aeruginosa* stimulate PMN chemotaxis [96,225]. Similarly, *N*-(3-oxododecanoyl)-l-homoserine lactone (3OC12-HSL), that controls virulence factor and biofilm formation in *P. aeruginosa*, has also been shown to induce PMN chemotaxis [226]. Interactions involving PMNs and such *P. aeruginosa* suspension aggregates are clinically highly relevant to CF airway disease and should be studied in great detail (Figure 1).

### 3.3. *P. aeruginosa* Resists NET-Mediated Attacks

The number of PMNs surrounding suspension aggregates in CF airways negatively correlates with bacterial growth rates in these aggregates [213]. These data suggest that PMNs limit *P. aeruginosa* growth in chronic CF, but instead of completely eliminating this bacterium, PMNs force *P. aeruginosa* to hide in the specialized environment of suspension aggregate cultures. The situation between PMNs and non-attached aggregates of *P. aeruginosa* can be considered as a compromise between the pathogen and the immune system. This view is somewhat new since the current opinion considers chronic CF airways as a hyperinflammatory environment. The presence of *P. aeruginosa* aggregates argues against this since bacteria found inside of these biofilms are nonmotile, nonvirulent showing a very slow metabolism. Several lines of evidence suggest that mucoid conversion and biofilm formation make *P. aeruginosa* resistant against most of the PMN antimicrobial effector mechanisms, including NET formation. Mucoid conversion of *P. aeruginosa* is a characteristic change over the course of CF lung infections and has been shown to prevent bacterial phagocytosis by macrophages and NET release from PMNs [227,228,229]. *P. aeruginosa* growing in biofilms activates the complement system less than planktonic forms [230]. *P. aeruginosa* LPS was suggested to be the main inducer of the classical pathway of complement activation by biofilms [230]. Biofilms trigger a strongly reduced respiratory burst in PMNs compared to *P. aeruginosa* planktonic forms [231]. *P. aeruginosa* bacteria residing in biofilms are able to respond to the presence of PMNs by inducing production of quorum sensing-controlled virulence factors, including rhamnolipids [232]. Rhamnolipids efficiently induce PMN lysis [233,234]. Induction of protective bacterial mechanism in response to PMNs supported a “launch a shield” model by which *P. aeruginosa* biofilms are surrounded by rhamnolipids that will eliminate immune cells including PMNs [43,232]. In support of this, inhibition of rhamnolipid synthesis in *P. aeruginosa* by inactivation of the rhamnolipid *rhlA* gene disabled bacterial protection against PMNs [235]. PMNs exhibiting some phagocytic activity have been observed on the surface of laboratory *P. aeruginosa* biofilms indicating that PMNs are capable of “chewing off” microbes from the biofilm surface [236,237]. Mucoid *P. aeruginosa* has also been reported to be fairly resistant against NET-mediated killing [170]. A main question remains how PMNs respond to suspension biofilms of *P. aeruginosa* (Figure 1).

### 3.4. Neutrophil Components Promote Biofilm Formation of *P. aeruginosa*

In addition to previous data indicating that *P. aeruginosa* is resistant in chronic CF airways against PMN effector mechanisms, several lines of evidence suggest that PMNs actually help biofilm growth of the bacterium. ecDNA is a main component of *P. aeruginosa* aggregates, and ecDNA obtained from necrotic PMNs has been shown to support aggregate growth of *P. aeruginosa* [214]. NET formation can likely be the source of PMN DNA in CF airways, since *P. aeruginosa* elicits robust NET release in PMNs [122,128,170,205]. Pyocyanin, a redox pigment and toxin of *P. aeruginosa*, is present in chronic CF airways and promotes the biofilm-forming ability of ecDNA [64,221]. Pyocyanin itself has also been shown to enhance NET release from human PMNs [130]. Whether PMNs respond to *P. aeruginosa* aggregates also with NET release remains an open question (Figure 1). If PMNs attempt to engulf bacterial suspension aggregates, NET extrusion could be their answer since according to a recent theory, PMNs preferentially release NETs in response to large particles that are impossible to phagocytose, including fungal hyphae and microcrystals [142,238,239] (Figure 1). PMNs have been shown to promote laboratory biofilm growth of *P. aeruginosa* in vitro [240,241,242]. Thus, *P. aeruginosa* not only becomes resistant against PMN effector mechanisms, but it uses PMNs to promote its biofilm growth in CF airways.

## 4. Conclusions

PMNs are well-equipped to eliminate *P. aeruginosa*. PMNs kill *P. aeruginosa* mainly intracellularly following phagocytosis. Neutrophils can also kill bacteria by releasing NETs or microvesicles. The proportion between intra- and extracellular killing mechanisms is crucial in determining the extents of PMN-mediated bacterial killing and collateral tissue damage in any disease. In immunocompromised individuals, such as CF patients, PMNs cannot clear *P. aeruginosa*. The interaction between neutrophils and *P. aeruginosa* is one of the most important features in CF airways. *P. aeruginosa* adapts to PMN-mediated attacks in CF by avoiding phagocytosis and forming resistant suspension microcolonies. PMNs are not capable of removing these suspension biofilms and release their dangerous antimicrobial cargo into the airway lumen to contribute to tissue damage. In summary, *P. aeruginosa* and PMNs engage in a complex, two-sided interaction in CF airways (Figure 1). It is essential to study its details to better understand CF airway inflammation and to design future neutrophil-based anti-inflammatory therapies in CF.
